# The neural basis of external responsiveness in prolonged disorders of consciousness

**DOI:** 10.1016/j.nicl.2019.101791

**Published:** 2019-03-26

**Authors:** Clara A. Stafford, Adrian M. Owen, Davinia Fernández-Espejo

**Affiliations:** a Brain and Mind Institute, Department of Psychology, The University of Western Ontario, London, Ontario N6C 5B7, Canada; b School of Psychology, The University of Birmingham, Edgbaston, Birmingham B15 2TT, United Kingdom; c Centre for Human Brain Health, University of Birmingham, Edgbaston, Birmingham B15 2TT, United Kingdom

**Keywords:** Coma, Diffusion tensor imaging, Motor control, Brain trauma, Disorders of consciousness

## Abstract

**Objective:**

To investigate the structural integrity of fibre tracts underlying overt motor behaviour in PDOC.

**Methods:**

This cross-sectional study examined 15 PDOC patients and 22 healthy participants. Eight PDOC patients met the criteria for the vegetative state, 5 met the criteria for the minimally conscious state and 2 met the criteria for emerging from the minimally conscious state. We used fibre tractography to reconstruct the white matter fibres known to be involved in voluntary motor execution (i.e., those connecting thalamus with M1, M1 with cerebellum, and cerebellum with thalamus) and used fractional anisotropy (FA) as a measure of their integrity.

**Results:**

PDOC patients showed significantly reduced FA relative to controls on the fibres connecting thalamus and M1. This went above and beyond a widespread injury to the white matter and correlated with clinical severity. In a subset of patients, we also identified a similar pattern of injury in the fibres connecting M1 and cerebellum but a relative preservation of those connecting cerebellum and thalamus.

**Conclusions:**

Our results suggest that structural damage to motor fibres may lead to reduced responsiveness in PDOC patients across all diagnostic sub-categories, and therefore behavioural assessments may underestimate the level of retained cognitive function and awareness across the PDOC spectrum.

## Highlights

• We assess the structural integrity of the fibres connecting the thalamus, primary motor cortex and cerebellum in PDOC
• PDOC patients showed reduced FA relative to controls on the fibres connecting thalamus and primary motor cortex
• Individual analyses in a subset of patients also revealed lower FA in supratentorial but not infratentorial tracts
• Structural damage to motor fibres may lead to reduced responsiveness in PDOC
• Behavioural assessments may underestimate the level of retained cognitive function

## 1 Introduction

The current gold-standard for diagnosing Prolonged Disorders of Consciousness (PDOC) is standardized behavioural scales (e.g., Coma Recovery Scale-Revised (CRS-R); Giacino and Kalmar, 2004), which require the patient to demonstrate their level of awareness through overt responses. PDOC refers to patients who remain in a state of wakefulness but absent or reduced awareness for >4 weeks (The Royal College of Physicians, 2013). Patients in the vegetative state (VS) show no overt behavioural signs of awareness and are thus considered to be unaware (Jennett and Plum, 1972; Laureys, 2005; The Multi-Society Task Force on PVS, 1994). Conversely, patients in the minimally conscious state (MCS) show inconsistent but reproducible signs of awareness (e.g., visual pursuit, command-following; Giacino et al., 2002; Giacino and Kalmar, 2005) and are considered to be at least minimally aware.

Nevertheless, recent studies have demonstrated that behavioural assessments, even by experienced teams, fail to diagnose a small group of VS patients, who show no signs of awareness externally but are able to show them *covertly* during functional neuroimaging tasks (Boly et al., 2007; Coleman et al., 2009; Fernández-Espejo and Owen, 2013; Owen et al., 2006). These typically involve motor imagery tasks (e.g., the patient is asked to imagine playing tennis; Fernández-Espejo and Owen, 2013; Monti et al., 2010) where the patients' neural responses are used as a proxy for volitional behavioural command-following; an ability that is clinically used as a measure of awareness.

We have previously provided a mechanistic explanation for the paradox between retained cognitive function and absent external responsiveness in covertly aware patients. We showed that structural damage to the fibres connecting the thalamus and the primary motor cortex (M1) may be disrupting an excitatory coupling between these structures, which is necessary for motor execution (but not motor imagery; Fernández-Espejo et al., 2015) Nevertheless, it remains unclear whether the covertly aware VS constitutes a separate diagnostic category or, in fact, damage to the motor system is contributing to reduced responsiveness (and an underestimation of patients' underlying cognitive capacities) in the broader PDOC group. Moreover, it is unknown whether the damage extends to other regions also crucial for motor execution beyond the thalamo-cortical tract. In fact, this tract is a sub-section of the larger dentato-thalamo-cortical path, via which the cerebellum exerts an inhibitory tone over M1 (Galea et al., 2009). In turn, M1 sends behaviourally relevant information to the cerebellum via the cortico-ponto-cerebellar tract, closing a loop that plays a critical role in voluntary motor control (Britsch et al., 1996; Lotze et al., 1999; Mattay et al., 1998; Nair et al., 2003), but crucially is not involved in motor imagery (Lotze et al., 1999; Nair et al., 2003).

We hypothesised that external responsiveness in PDOC would correlate with the structural integrity of the tracts connecting the ventrolateral thalamus (VL) to M1 (henceforth referred to as the *thalamo-cortical tract*) across different diagnostic categories, and that damage to the motor network may extend to the tracts connecting the dentate nucleus (DN) to the VL (henceforth referred to as the *cerebello-thalamic tract*) and M1 to the DN (henceforth referred to as the *cortico-cerebellar tract*). To test these hypotheses, we conducted a cross-sectional diffusion tensor tractography study.

## 2 Methods

### 2.1 Participants

As part of a broader research program at the University of Western Ontario (UWO), we recruited a convenience sample of 15 PDOC patients (8 male, aged 19–57, *M* = 37.33, *SD* = 12.25) between 2012 and 2015 (8 VS, 5 MCS, 2 emerging from MCS [EMCS]). Exclusion criteria included lack of eligibility for the MRI environment. See Table 1, Table 2 for a summary of clinical and demographic information. Previous studies from our group reported functional (Beukema et al., 2016; Fiacconi and Owen, 2016; Gibson et al., 2014, Gibson et al., 2016; Naci and Owen, 2013) or structural (Fernández-Espejo et al., 2015; Lant et al., 2016) datasets for subsets of this patient cohort.

**Table 1:** Patient demographic and clinical information.

Patient ID	Age	Sex	Diagnosis (CRS-R^a^)	Aetiology^b^	TPI^c^ (years)
VS1	38	M	VS (7)	TBI	12
VS2⁎	27	M	VS (8)	TBI	7
VS3	44	F	VS (7)	TBI	20
VS4	57	M	VS (7)	Anoxic	3
VS5	20	F	VS (8)	Other	6
VS6	19	M	VS (7)	Anoxic	2 months
VS7⁎	51	F	VS (5)	Anoxic	1
VS8⁎	52	F	VS (6)	Anoxic	6
MCS1	33	M	MCS (10)	Anoxic	15
MCS2	46	F	MCS (10)	Anoxic	19
MCS3	27	M	MCS (13)	Anoxic	3
MCS4⁎	25	F	MCS (9)	TBI	6
MCS5⁎	40	M	MCS (8)	TBI	3
EMCS1⁎	49	F	EMCS (22)	TBI	12
EMCS2	32	M	EMCS (23)	TBI	4
Mean	37.33				7.81
SD	12.25				6.32

a Total CRS-R score based on the patient's highest performance in each of the sub-scales.

b TBI = traumatic brain injury.

c TPI = time post-ictus.

⁎ Patients included in the closed-loop analyses.

**Table 2:** Patient behavioural scores from the CRS-R.

Scale	Patient ID								
	VS1	VS2^a^	VS3	VS4	VS5	VS6	VS7^a^	VS8^a^	MCS1
Auditory function									
4-Consistent movement to command^b^	–	–	–	–	–	–	–	–	–
3-Reproducible movement to command^b^	–	–	–	–	–	–	–	–	–
2-Localization to sound	–	2	–	–	2	2	–	–	–
1-Auditory startle	1	–	1	1	–	–	1	1	1
0-None	–	–	–	–	–	–	–	–	–

Visual function									
5-Object Recognition^b^	–	–	–	–	–	–	–	–	–
4-Object localization: Reaching^b^	–	–	–	–	–	–	–	–	–
3-Visual pursuit^b^	–	–	–	–	–	–	–	–	3
2-Fixation^b^	–	–	–	–	–	–	–	–	–
1-Visual startle	1	1	1	1	1	1	1	–	–
0-None	–	–	–	–	–	–	–	–	–

Motor function									
6-Funtional object use^b^	–	–	–	–	–	–	–	–	–
5-Automatic motor response^b^	–	–	–	–	–	–	–	–	–
4-Object manipulation^b^	–	–	–	–	–	–	–	–	–
3-Localization to noxious stimulation^b^	–	–	–	–	–	–	–	–	–
2-Flexion withdrawal	2	2	2	2	2	–	–	2	2
1-Abnormal posturing	–	–	–	–	–	1	1	–	–
0-None/Flaccid	–	–	–	–	–	–	–	–	–

Oromotor/verbal function									
3-Intelligible verbalization^b^	–	–	–	–	–	–	–	–	–
2-Vocalization/oral movement	–	–	–	–	–	–	–	–	–
1-Oral reflexive movement	1	1	1	1	1	1	1	1	1
0-None	–	–	–	–	–	–	–	–	–

Communication									
2-Functional: Accurate^c^	–	NA	–	NA	–	–	–	–	–
1-Non-functional: Intentional^b^	–	NA	–	NA	–	–	–	–	–
0-None	–	NA	–	NA	–	–	–	–	0

Arousal									
3-Attention	–	–	–	–	–	–	–	–	3
2-Eye opening w/o stimulation	2	2	2	2	2	2	–	2	–
1-Eye opening w/ stimulation	–	–	–	–	–	–	1	–	–
0-Unarousable	–	–	–	–	–	–	–	–	–
Total CRS-R score	7	8	7	7	8	7	5	6	10


Scale	Patient ID					
	MCS2	MCS3	MCS4^a^	MCS5^a^	EMCS 1^a^	EMCS 2
Auditory function						
4-Consistent movement to command^b^	–	–	–	–	4	4
3-Reproducible movement to command^b^	–	3	–	–	–	–
2-Localization to sound	2	–	2	–	–	–
1-Auditory startle	–	–	–	1	–	–
0-None	–	–	–	–	–	–

Visual function						
5-Object Recognition^b^	–	–	–	–	5	5
4-Object localization: Reaching^b^	–	–	–	–	–	–
3-Visual pursuit^b^	3	3	3	3	–	–
2-Fixation^b^	–	–	–	–	–	–
1-Visual startle	–	–	–	–	–	–
0-None	–	–	–	–	–	–

Motor function						
6-Funtional object use^b^	–	–	–	–	6	6
5-Automatic motor response^b^	–	–	–	–	–	–
4-Object manipulation^b^	–	–	–	–	–	–
3-Localization to noxious stimulation^b^	–	–	–	–	–	–
2-Flexion withdrawal	2	2	–	–	–	–
1-Abnormal posturing	–	–	1	1	–	–
0-None/Flaccid	–	–	–	–	–	–

Oromotor/verbal function						
3-Intelligible verbalization^b^	–	–	–	–	–	3
2-Vocalization/oral movement	–	2	–	–	2	–
1-Oral reflexive movement	1	–	1	1	–	–
0-None	–	–	–	–	–	–

Communication						
2-Functional: Accurate^c^	–	–	–	–	2	2
1-Non-functional: Intentional^b^	–	–	–	–	–	–
0-None	–	–	–	–	–	–

Arousal						
3-Attention	–	3	–	–	3	3
2-Eye opening w/o stimulation	2	–	2	2	–	–
1-Eye opening w/ stimulation	–	–	–	–	–	–
0-Unarousable	–	–	–	–	–	–
Total CRS-R score	10	13	9	8	22	23

NA: Could not be evaluated.

a Included in both analyses.

b Denotes MCS.

c Denotes emergence from MCS.

We also recruited a cohort of 22 right-handed volunteers (12 males, aged 18–29; *M* = 23.86; *SD* = 3.211), with no history of psychiatric or neurological disorders, between 2011 and 2014. From this group, 2 volunteers were scanned with 2 different sequences (see MRI Acquisition below). This resulted in 22 healthy volunteers but 24 healthy diffusion datasets.

All healthy controls gave written informed consent and were paid for their participation in the experiment. Surrogate decision makers provided written assent for all patients. The Health Sciences Research Ethics Board of UWO provided ethical approval for these studies.

### 2.2 MRI acquisition

We acquired MRI data at the Centre for Functional and Metabolic Mapping (CFMM), Robarts Research Institute (London, Canada). The CFMM upgraded their 3 T scanner during participant recruitment and developed a more advanced diffusion tensor imaging (DTI) protocol. Fourteen participants (7 patients, 7 healthy volunteers) were scanned pre-upgrade with a 64-direction protocol in a Magnetom Trio system (Siemens, Erlanger, Germany): b-value = 700 s/mm^2^, TR = 8700 ms, TE = 77 ms, voxel size = 2x2x2mm, no gap, 77 slices (6:19 min). Four participants (2 patients, 2 healthy volunteers) were scanned with the same sequence in a Prisma Magnetom Trio system (Siemens, Erlanger, Germany), after the upgrade. Finally, these 2 healthy volunteers, alongside 13 new healthy volunteers and 6 patients were scanned with a 137-directions protocol in the Prisma system: b-value = 1500s/mm^2,^ TR = 1980 ms, TE = 71 ms, voxel size = 2x2x2mm, no gap, 64 slices, multiband acceleration factor = 4, acquired over two phase-encoding directions (4:45 min each). This resulted in a balanced distribution of patients (*n* = 15) and healthy controls (n = 15) across the two different scanners/sequences for the analyses involving the thalamo-cortical tract (Note that after inspection of the diffusion data -see Statistical Analyses below- only 11 PDOC patients were included in the group analyses) and a representative ‘healthy’ sample (n = 15) with 137-direction data to compare each of the 6 individual patients who were also scanned with this sequence to in the analyses of all 3 tracts (i.e., single-subject analyses).

### 2.3 DTI analyses

We processed all images using FMRIB Software Library (FSL) (http://fsl.fmrib.ox.ac.uk/fsl/fslwiki/) (Smith et al., 2004), following a similar pipeline as in our previous studies (Fernández-Espejo et al., 2012, Fernández-Espejo et al., 2015; Lant et al., 2016). Pre-processing steps included correction for eddy-current distortions and head movement (Andersson and Sotiropoulos, 2016), skull and non-brain tissue stripping (Brain Extraction Tool; Smith, 2002) and local tensor fitting (FMRIB Diffusion Toolbox). For the Prisma sequence, we also used topup (Andersson et al., 2003; Smith et al., 2004) to estimate the susceptibility-induced off-resonance field from the pairs of images that resulted from the reversed phase-encode blips used in data collection. Note that it was not possible to apply topup in the 64-direction dataset as this was acquired over a single phase-encoding direction.

### 2.4 Region of interest masking

We used 3 regions of interest (ROIs): bilateral VL, M1, and DN, generated as spherical masks using fslmaths, We created M1 masks as 6-mm radius spheres centered in MNI space at coordinates previously reported by Fernández-Espejo and colleagues (Fernández-Espejo et al., 2015). We then un-warped these masks to each participant's native space. For this, we extracted the b0 image for each participant and normalised it into MNI space using affine linear registration (FLIRT; Jenkinson et al., 2002; Jenkinson and Smith, 2001), and then we inverted the resulting normalisation parameters and applied them to our original M1 mask using the FLIRT tools *InvertXFM* and *ApplyXFM*. Similar to previous studies (Fernández-Espejo et al., 2011; Lant et al., 2016), this method led to accurate results for cortical masks but resulted in registration errors in patients when used to obtain subcortical masks, due to the severe damage and atrophy in these areas. Therefore, we manually identified the coordinates for both subcortical masks in each individual participant. Specifically, we generated a 4-mm sphere VL mask centered 6-mm dorsal to the anterior commissure, based on the stereotactic atlas by Morel (Morel, 2007). We used fractional anisotropy (FA) and axial plane vector (V1) RGB colour maps to ensure our sphere fell within the borders of the ventrolateral nuclei. For the DN masks, we used the SUIT atlas template of the human cerebellum and brainstem (Diedrichsen, 2006) as a visual reference and generated a 6-mm sphere centered in the region where Vermis X begins to protrude into the 4th ventricle.

Additionally, for each participant, we manually created a number of masks to use as termination or exclusion masks during tract reconstruction (see Connectivity Analysis below). For the reconstruction of the thalamo-cortical tracts, we used an inter-hemispheric mask to stop fibres crossing to the contra-lateral hemisphere, and a subthalamic mask to stop fibres travelling from thalamus to brainstem. For the reconstruction of the cerebello-thalamic tract, we used a mask placed at each superior cerebellar peduncle to exclude fibres that crossed to the contralateral cerebellar hemisphere. Finally, for the cortico-cerebellar tract, we used a supra-tentorial inter-hemispheric mask to exclude fibres crossing hemispheres through the corpus callosum, as well as the superior cerebellar peduncles masks described above.

### 2.5 Connectivity analysis

We performed fibre tracking between ROIs in diffusion native space for each subject, using probtrackX (Behrens et al., 2003; Behrens et al., 2007). We tracked 5000 streamlines per voxel from seed to target and then from target to seed, averaged the resulting probability distributions, and thresholded them to 2% of the maximum number of streamlines for each subject for both the thalamo-cortical and cortico-cerebellar tracts. For the cerebello-thalamic tracts, this threshold resulted in the inclusion of many anatomically implausible fibres and therefore, we used a more conservative 5% threshold (Behrens et al., 2007; Fernández-Espejo et al., 2012; Kinoshita et al., 2015; Sala-Llonch et al., 2010). We selected each threshold after careful inspection of all tracts in each individual participant by one of the authors (C.A.S.), and in order to maximise the inclusion of anatomically viable fibres and the removal of false positives across subjects for each tract. Note that we carried out this process before extracting any FA values or performing any statistical analyses. While there is currently no standard value for thresholding tracts, values between 2 and 5% have proven successful in previous studies in this patient population (Lant et al., 2016; Xu et al., 2007; Zheng et al., 2017).

We could successfully reconstruct thalamo-cortical tracts in all controls and all but 3 patients (see below). However, the 64-direction sequence did not allow for reliable reconstruction of the cerebello-thalamic or cortico-cerebellar fibres in either healthy controls or patients. Specifically, this sequence failed to solve the crossing fibres of the middle cerebellar peduncles or the decussation of the superior cerebellar peduncles, which resulted in anatomically implausible reconstructions. This is likely due to the reduced number of directions, lower b-value, and the acquisition of a single phase-encoding direction in the 64-direction sequence as compared to the 137-directions one which are known to greatly impair the sensitivity to reconstruct complex white matter structures (Fan et al., 2014; Heiervang et al., 2006). Therefore, analyses referring to these 2 paths were only performed in the subset of 6 patients and 15 healthy participants who had data with the 137-direction sequence acquired in the Prisma system.

### 2.6 Statistical analyses

#### 2.6.1 Thalamo-cortical tracts

We calculated the mean FA value of each tract in each participant using fslmaths. We were unable to reconstruct the thalamo-cortical tracts MCS1, VS5, and VS8, and therefore we excluded them from the group analyses. Additionally, we excluded VS7 as extreme value analysis revealed their FA was >3 standard deviations away from the mean, and scores beyond this point have a cumulative probability of <0.2% (Aggarwal, 2015).

In order to assess and control for the effects of global white matter damage, global FA was calculated in all participants by thresholding the FA map to 0.2 and calculating the mean FA values of the remaining voxels above this threshold.

We performed frequentist and equivalent Bayesian comparisons (with default priors) with IBM SPSS Statistics v.23, and JASP v.0.8.6 (2018) respectively. Specifically, we compared patients and healthy controls (*n* = 15, aged 18 to 29 years, *M* = 24.73, *SD* = 2.68, 8 males) with a repeated measures ANCOVA using hemisphere as within-subject factor and group (healthy controls/PDOC) as the between-subject factor. We performed linear correlations between the dependent variables (i.e., left and right thalamo-cortical tracts), age, sequence combination (i.e., 64-directions Trio, 64-directions Prisma, and 137-directions Prisma), and global FA value to determine which of these variables should be included as covariates of no-interest. Only FA and age showed significant correlations and were included in the ANCOVA.

As we were interested in the relationship between damage to thalamo-cortical fibres and the patient's ability to show external signs of awareness, we ran an ANCOVA grouping patients based on their capabilities to respond behaviourally during clinical assessments: *behaviourally unresponsive* (VS; *n* = 6), versus *behaviourally responsive* (MCS + EMCS; *n* = 6) and using hemisphere as a within-subject factor. Age, time post-ictus, aetiology, sequence combination, and global FA did not correlate with the dependent variables and therefore were not included in the ANCOVA.

In both cases, we performed post-hoc independent samples *t*-tests when significant main effects and/or interactions were identified. Significance was set at a *p* < .05.

Additionally, a Jeffrey-Zellner-Siow Bayes factor (JZS-BF_10_) contrasted the strength of the evidence for models reflecting the null, main effects, and interactions (Rouder et al., 2012). Bayes factors allow to evaluate the evidence for both the presence and the absence of an effect in a non-dichotomous way (reject or fail-to-reject), by comparing how different models explain the data given the factors of interest (Raftery, 1995; Rouder et al., 2017). To obtain the evidence for our effect of interest (i.e., group or responsiveness), we compared a model including this effect plus the relevant nuisance variables (as determined by the correlations described above) with a model including only nuisance variables. Additionally, we calculated a Bayes factor for the inclusion of the variable of interest (BF_inclusion_) by comparing all models that include this variable with all models that do not. A JSZ-BF_10_ between 1/3 and 3 is considered to be weak/anecdotal evidence for an effect; 3–10: substantial evidence; 10–100: strong evidence; >100: very strong evidence (Jeffreys, 1961).

Finally, we performed Spearman's rank-order correlations to investigate the relationship between a patient's CRS-R score and FA in the left and right tracts.

#### 2.6.2 Closed loop: Thalamo-cortical, cortico-cerebellar, cerebello-thalamic tracts

Due to the reduced sample size (6 patients) we were not able to perform group statistics. Instead, we compared data from each individual patient to a representative healthy control group (*n* = 15, aged 18 to 29 years, *M* = 23.80, *SD* = 3.34, 7 males) using Crawford's Modified t-tests (Crawford et al., 2011). As we used 3 masks (for each hemisphere) to reconstruct 3 segments of a closed loop, we expected some voxels to be included in 2 or more of the reconstructed tracts. To ensure no voxel was included in more than one analysis, we limited our calculations to the voxels that were unique to each tract. Specifically, we subtracted the voxels included in the thalamo-cortical segment from the cerebello-thalamic tract, and the voxels included in thalamo-cortical and cerebello-thalamic segments from the cortico-cerebellar tract before calculating FA values. We hypothesised that patients' tracts would have lower FA than the healthy control's mean and treated each patient as an independent test, and thus we established significance at one-tail Bonferroni corrected *p* = .0083. The patient group included 3 VS, 2MCS, and 1 EMCS patients (2 male, aged 25–52, *M* = 40.67, *SD* = 12.14). See Table 1 and eMethods for a more detailed clinical history.

## 3 Results

### 3.1 Thalamo-cortical tracts

**Fig. 1:**
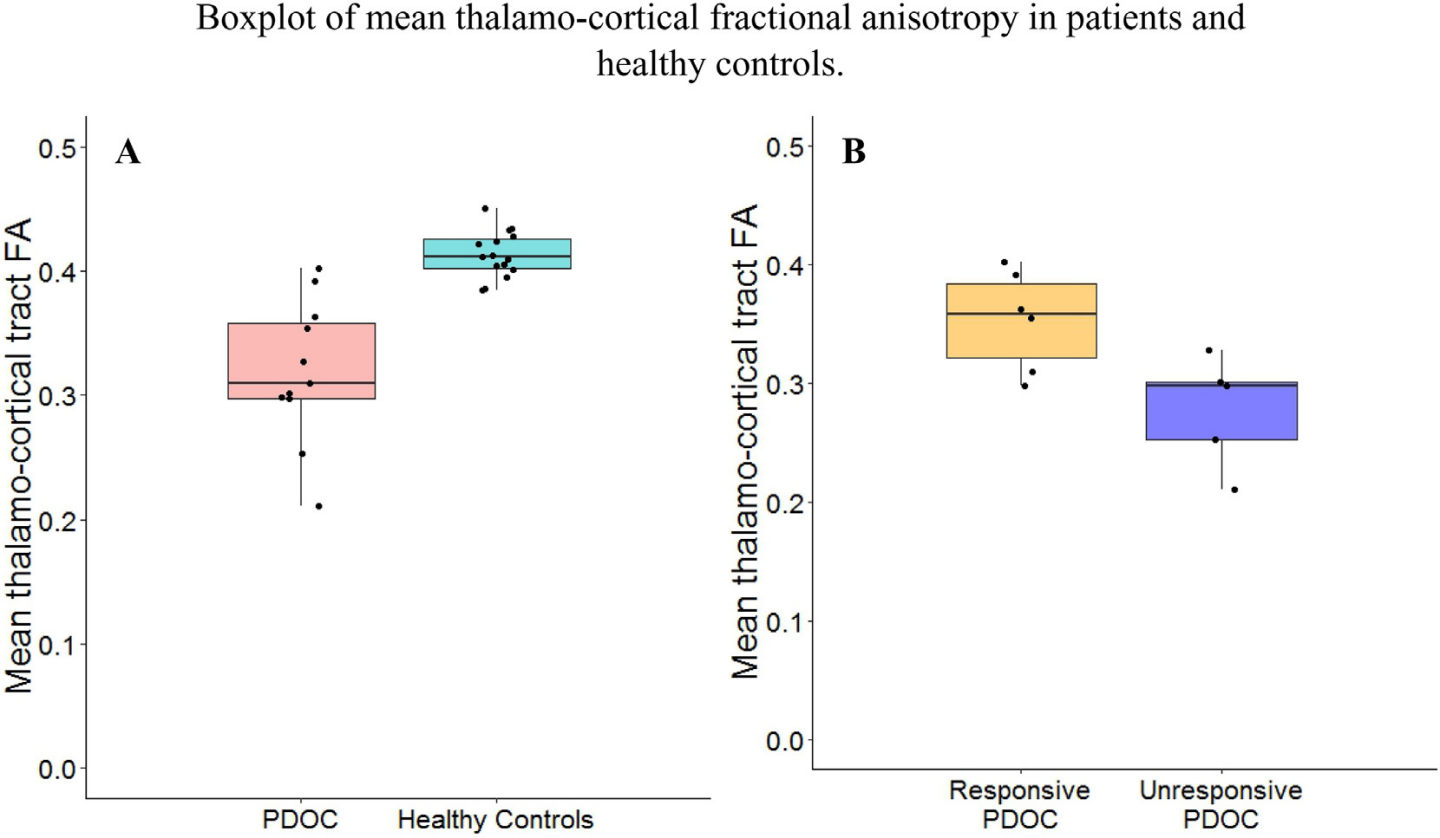
Panel A: Mean thalamo-cortical tract fractional anisotropy in PDOC patients and healthy controls; Panel B: Mean thalamo-cortical tract fractional anisotropy in responsive and unresponsive patients. Middle lines represent the medians; Hinges correspond to the first and third quartiles; Whiskers extend from the hinges to the largest and smallest values. Boxplots are overlaid with participant data points (black dots).

Repeated measures ANCOVA, with age and global FA as covariates, revealed significantly lower FA in PDOC as compared to healthy controls (F_1,22_ = 10.888, *p* = .003). No significant effect of hemisphere, age or global FA was detected. A Bayesian equivalent showed strong support for an effect of group (JSZ-BF_10_ = 68.8 for a model including group, age, and global FA relative to the model including age and global FA only; group BF_inclusion_ = 81.475) and substantial evidence for the effect of an interaction between hemisphere and group (BF_inclusion_ = 3.364). See Fig. 1.

There were no significant differences in time-post ictus (*t*_9_ = −0.188, *p* = .855, JSZ-BF_10_ = 0.486) or age (*t*_9_ = −0.067, *p* = .948, JSZ-BF_10_ = 0.481) between responsive and unresponsive patients. Repeated measures ANCOVA revealed that unresponsive patients had significantly lower FA than responsive patients (F_1,9_ = 8.007, *p* = .02) for this tract. The Bayesian equivalent also showed substantial evidence for the effect of responsiveness as compared to all other models (JSZ-BF_10_ = 3.534 for the model including responsiveness and hemisphere relative to the model including hemisphere only; responsiveness BF_inclusion_ = 3.586). See Fig. 1.

Additionally, when comparing each subgroup to healthy controls, we observed significant differences in FA both in responsive (*F*_1,17_ = 5.231, *p* = .035) and unresponsive patients (*F*_1,16_ = 47.429, *p* < .001). For the latter, we also observed a significant effect of Age (*F*_1,16_ = 6.967, *p* = .018). Similarly, the Bayesian equivalent indicated strong evidence for the effect of Group as compared to all other models when comparing responsive patients to healthy controls (JSZ-BF_10_ = 7.617 for the model including group, age, and global FA relative to the model including age and global FA only; group BF_inclusion_ = 15.603). However, when comparing unresponsive patients to healthy controls, while there was very strong evidence for the effect of group (JSZ-BF_10_ = 3372.782 for the model including group, age, and global FA relative to the model including age and global FA only; group BF_inclusion_ = 20,972.504), there was also substantial evidence for the effect of age (BFi_nclusion_ = 4.177) and strong evidence for the interaction between hemisphere and group (BF_inclusion_ = 13.352).

On the basis of this effect, we conducted follow-up analyses separately for the left and right hemispheres. On the left hemisphere, our Bayesian ANCOVA showed very strong evidence for the effect of group, as compared to all other models (JSZ-BF_10_ = 863.104 for the model including group, global FA and age relative to the model including global FA and age only; group BF_inclusion_ = 869.676). In contrast, while our analyses also indicated a very strong effect of group on the right hemisphere (JSZ-BF_10_ = 13,958.967 for the model including group, global FA and age relative to the model including global FA and age only; group BF_inclusion_ = 8309.276), there was substantial evidence for the added effect of global FA (BF_inclusion_ = 3.036) and weak evidence for the added effect of Age (BF_inclusion_ = 2.608).

Finally, CRS-R scores were significantly correlated with the left thalamo-cortical tract (rho = 0.769, *p* = .006) and correlated at trend-level with the right thalamo-cortical fibre tract (rho = 0.578, *p* = .062).

### 3.2 Single-patient analyses: Closed loop

**Fig. 2:**
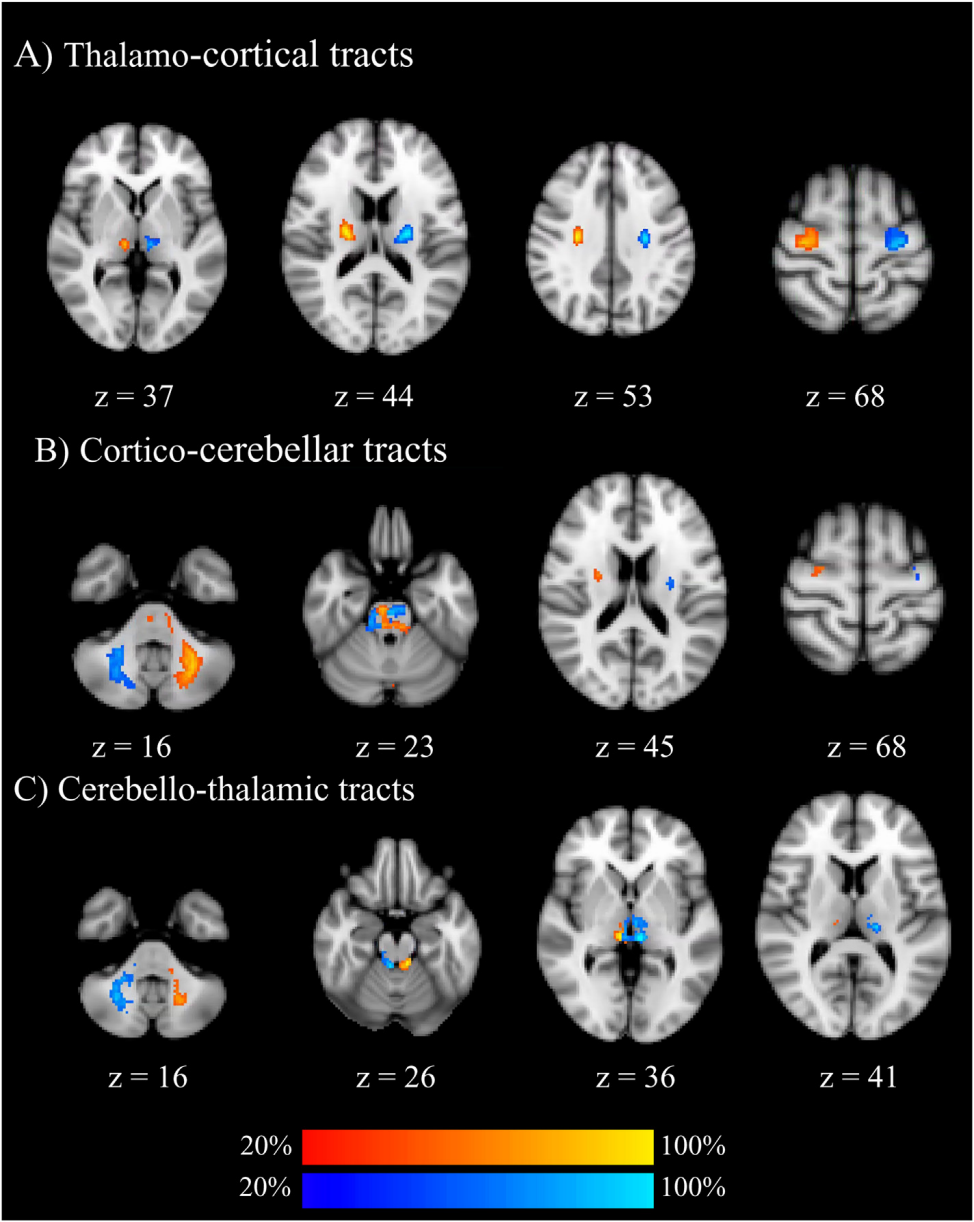
Group probability maps of reconstructed tracts in healthy controls using the 137 directions diffusion sequence. Maps are thresholded at presence in at least 20% of healthy subjects. Tracts in red involve the right ventrolateral thalamus and tracts in blue involve the left ventrolateral thalamus. Images are displayed in Montreal Neurological Institute standard stereotaxic space (2 mm), and coordinates are provided for each slice. (For interpretation of the references to color in this figure legend, the reader is referred to the web version of this article.)

Patient VS2 showed significantly lower FA on both thalamo-cortical tracts and on the left cortico-cerebellar tract. Patient VS7 showed significantly reduced FA in all tracts except the cerebello-thalamic ones. In patient VS8, we were unable to reconstruct the thalamo-cortical and cortico-cerebellar tracts; they showed an FA reduction in the right cerebello-thalamic tract. Patient MCS4 showed no significant FA reductions in any of the tracts. Patient MCS5 showed bilateral FA reductions on both thalamo-cortical tracts and cortico-cerebellar tracts. Finally, patient EMCS1 showed significantly reduced FA on their right thalamo-cortical and right cortico-cerebellar tracts. See Table 3 and Fig. 2.

**Table 3:** *P*-values, point estimates and effect sizes of patient FA values based on Crawford's modified t-test.

Patient	Fibre tract	*P-*value	Effect size
VS2	Left VL-M1	0.0005558⁎⁎	−4.220
	Right VL-M1	0.0000002⁎⁎	−9.247
	Left M1-DN	0.0000751⁎⁎	−5.307
	Right M1-DN	ns	–
	Left VL-DN	ns	–
	Right VL-DN	ns	–
VS7	Fibre tract	*P-*value	Effect size
	Left VL-M1	0.0000000⁎⁎	−12.960
	Right VL-M1	0.0000000⁎⁎	−15.444
	Left M1-DN	0.0000424⁎⁎	−5.631
	Right M1-DN	0.0006330⁎⁎	−4.151
	Left VL-DN	ns	–
	Right VL-DN	ns	–
VS8	Fibre tract	*P-*value	Effect size
	Left VL-M1	NA	–
	Right VL-M1	NA	–
	Left M1-DN	NA	–
	Right M1-DN	NA	–
	Left VL-DN	ns	−1.844
	Right VL-DN	0.0012831⁎	−3.781


	Fibre tract	*P-*value	Effect size
MCS4	Left VL-M1	ns	–
	Right VL-M1	ns	–
	Left M1-DN	ns	–
	Right M1-DN	ns	–
	Left VL-DN	ns	–
	Right VL-DN	ns	–
MCS5	Fibre tract	*P-*value	Effect size
	Left VL-M1	0.0011719⁎	−3.828
	Right VL-M1	0.0000706⁎⁎	−5.341
	Left M1-DN	0.0000240⁎⁎	−5.963
	Right M1-DN	0.0055660⁎	−3.018
	Left VL-DN	ns	–
	Right VL-DN	ns	–
EMCS1	Fibre tract	*P-*value	Effect size
	Left VL-M1	ns	–
	Right VL-M1	0.0008911⁎⁎	−3.971
	Left M1-DN	ns	
	Right M1-DN	0.0001628⁎⁎	−4.878
	Left VL-DN	ns	–
	Right VL-DN	ns	–

NA: tract not available for the analysis; ns: non-significant difference.

NB. We only analysed tracts in which the FA was reduced as compared to healthy controls.

⁎ significant at 1-tail Bonferroni corrected *p* < .0083.

⁎⁎ significant at *p* < .001.

## 4 Discussion

Our data suggest that structural damage to the pathways that support external motor behaviour may be interfering with PDOC patients' ability to demonstrate their ‘true’ level of awareness across multiple diagnostic groups. We previously demonstrated that the ability to follow commands behaviourally relies on successful excitatory input from thalamus to M1, and that structural damage to the white matter fibres connecting these two regions may explain the lack of external responsiveness in covertly aware VS patients (Fernández-Espejo et al., 2015). Our current results expand our conclusions in two important ways: First, they suggest that the covertly aware VS may simply represent the most extreme case of misdiagnosis due to a cognitive-motor dissociation (Schiff, 2015). Therefore, standard clinical assessments may underestimate the *true* level of awareness in the broader PDOC group. Second, they indicate that damage to other pathways within the motor network, beyond thalamo-cortical fibres, may also contribute to the lack of responsiveness in PDOC.

Specifically, we identified significant damage to the fibres connecting thalamus and M1 in PDOC patients, which went above and beyond a widespread injury to the whole-brain white matter tissue. Importantly, while both behaviourally responsive and behaviourally unresponsive patients showed such damage, this was significantly more severe in the latter. Indeed, our individual analyses of a sub-set of patients revealed severe bilateral damage to this tract in all VS patients, 1 out of 2 MCS patients, and only right unilateral damage in our EMCS patient. This is consistent with previous histopathological and imaging reports of neuronal loss in the motor cortex (William L. Maxwell et al., 2010) and thalamus (Fernández-Espejo et al., 2010; Lutkenhoff et al., 2015; W. L. Maxwell et al., 2004; Zheng et al., 2017), the severity of which increases with the severity of the PDOC. In traumatic patients (Fernández-Espejo et al., 2010; Lutkenhoff et al., 2015; Maxwell et al., 2004; Maxwell et al., 2010), damage to these structures could be the result of post-traumatic transneuronal degeneration following disconnections caused by the diffuse axonal injury. Nevertheless, in lack of longitudinal studies that identify primary and secondary injuries, the specific mechanisms that relate white and grey matter damage in PDOC patients can only be speculated upon.

Interestingly, we identified lateralisation effects in the damage to the thalamo-cortical tract. In unresponsive patients, damage to the right tract seemed to be related to patients' widespread damage to the whole-brain white matter tissue, while damage to the left tract showed greater specificity to explain the differences between these patients and healthy controls. Moreover, damage to the left hemisphere, but not the right, was correlated with clinical severity, as estimated by CRS-R scores in the full PDOC group. This is consistent with previous findings of left-lateralised thalamic (Fernández-Espejo et al., 2010; Lutkenhoff et al., 2015) and global grey matter (Guldenmund et al., 2016) atrophy in PDOC. Others (Guldenmund et al., 2016; Lutkenhoff et al., 2015) have argued that this left lateralisation may be related to deficits in linguistic processing and highlight the dependence of standard clinical assessments of consciousness on this function. In contrast, we speculate that the previously reported thalamic atrophy may have been at least partially related to damage in motor thalamo-cortical paths, and that the identified left lateralisation could be reflecting the role of hemispheric dominance on motor deficits after brain injury, and thus highlighting the reliance on motor responsiveness of behavioural scales for the assessment of consciousness. Indeed, early studies in patients with focal brain injuries suggest that left hemispheric damage results in a more severe deficit in motor functioning as compared to right hemispheric damage (Kimura, 1977; Wyke, 1966, Wyke, 1967, Wyke, 1968; York Haaland and Harrington, 1989). In the healthy brain, handedness can influence the asymmetry of white matter bundles to different degrees across brain regions or networks (Davidson and Hugdahl, 1996). In the motor system specifically, right-handed individuals seem to have increased structural connectivity in the left motor cortex (Amunts et al., 1996) and a larger number of fibres in the left corticospinal tract (Herve et al., 2009). In contrast, left-handers do not typically show the reversed pattern and are instead characterised by a less marked asymmetry of the left hemisphere (Amunts et al., 1996; Herve et al., 2009). The functional implications of this asymmetry are not entirely understood. Some studies have reported that the right motor cortex shows preferential activation to contralateral finger movements, while the left motor cortex also shows activity during ipsilateral ones; specially in right handed individuals (Kim et al., 1993). This may explain why lesions of the left hemisphere result in a greater motor dysfunction (Wyke, 1968; York Haaland and Harrington, 1989). However, more recent studies have found no functional asymmetries in the motor system nor a relationship between the laterality of its functional connections and handedness (e.g., Nielsen et al., 2013). It is worth noting that we could not verify handedness in our patients and therefore any discussion about hemispheric dominance in our patients remains necessarily speculative. By definition, it is not possible to directly assess handedness in non-responsive patients and family reports are often unreliable and/or difficult to obtain. As a result, this variable is rarely reported in the PDOC literature. Moreover, we had not hypothesised a laterality effect in our study that granted a formal investigation of patient handedness, and therefore, future studies are needed before we can make strong conclusions about this.

Additionally, our analysis of the structural integrity of a closed motor loop in a subset of patients revealed an increase in the extent of the damage that goes along with clinical severity. Specifically, VS patients tended to exhibit extensive damage across the thalamo-cortical and cortico-cerebellar segments of the loop. In contrast, only one responsive patient (MCS5) showed such pattern of damage. Our own research (Fernández-Espejo et al., 2015) and early trace studies (Jakab et al., 2012; Rausell and Avendaño, 1985; Rispal-Padel et al., 1973) demonstrated that the thalamo-cortical tract is necessary for voluntary motor command-following. In turn, the cortico-cerebellar pathway allows the cerebellum to use information from the motor cortex to anticipate the consequences of actions and regulate motor activity via its projections to the thalamus (Lawrenson et al., 2018; Purves et al., 2001). It is therefore plausible that damage at any point of this loop may result in a disruption of the excitatory thalamo-cortical output that is necessary for overt command following, and lead to reduced external responsiveness as a result.

Interestingly, cerebello-thalamic fibres appeared relatively preserved across the group, with only unilateral damage in one VS patient, and no differences from controls in the rest. This may explain the previously reported partial preservation of functional connectivity between thalamus and cerebellum at rest in PDOC (Zhou et al., 2011). The structural integrity of the cerebellum in PDOC has been relatively understudied. Nonetheless, our results agree with early reports of cerebellar atrophy in some traumatic (Guldenmund et al., 2016) and non-traumatic patients (Adams et al., 2000; Juengling et al., 2005), which did not correlate with clinical severity and were inconsistent even in VS patients. Importantly, the cerebellum exerts an inhibitory tone over the thalamus (Galea et al., 2009) and, therefore, the relative preservation of cerebello-thalamic fibres may result in excessive thalamic inhibition that may further contribute to the reduced responsiveness in PDOC.

It is worth noting that our sample included both traumatic and non-traumatic cases. Our results thus suggest that damage to the motor system is related to responsiveness across both aetiological groups. However, our sample size precluded us from performing formal comparisons between them and further research is needed to elucidate whether aetiological differences lead to distinct patterns of white matter injury in the motor loop.

Several limitations must be considered when interpreting the results of this study. First, our group analyses included data acquired with different combinations of sequence and scanner, and slight differences in the preprocessing pipeline for each sequence (specifically in the correction for eddy currents and susceptibility-induced distortions). To minimize the effects of these variables, we carefully matched patients and healthy volunteers to ensure we had the same distribution of sequence combinations in each group. Moreover, our statistical analyses suggested that neither sequence nor scanner had an effect in our dependent variables, and therefore it is unlikely they may have influenced our effects. Furthermore, previous literature has shown that larger white matter tracts, such as those included in this study, show little coefficient variability across different sequences and scanners (Palacios et al., 2017). Nevertheless, our results should be interpreted with caution. Second, it is important to note the heterogeneous time post-ictus in our PDOC group. While most of our patients had been in these conditions for several years, patient VS6 was only 2-months post-ictus. However, and crucially, none of our analyses indicated a significant effect of time post-injury. Moreover, a re-analysis excluding patient VS6 performed during the revision process confirmed our original effects, suggesting that the results reported here are not affected by this variable. Finally, our study included a small sample of PDOC patients. Nevertheless, we confirmed our main effects with both frequentist and Bayesian methods, and the latter are typically well equipped to model small sample data. Notwithstanding this, the estimates in Bayesian statistics are highly sensitive to the specification of priors (Van De Schoot et al., 2015). There is no previous literature on the structural integrity of motor fibres in PDOC that could inform our definition of priors, and therefore we used *default* priors based on desirable theoretical properties of the Bayes factor, and as developed by Rouder and colleagues (Rouder et al., 2012). While this typically offers an adequate solution in cases where the literature is scarce, our study can further inform the definition of the prior distribution in future studies aimed at replicating our findings in a larger group of patients.

## 5 Conclusions

We demonstrated that structural damage to the fibres connecting the thalamus with M1 is related to reduced external responsiveness in PDOC across all diagnostic categories. Furthermore, we showed that this damage extends to other tracts also crucial for the voluntary control of movement. Overall, our results suggest that behavioural assessments may underestimate the level of retained cognitive function and awareness for all sub-categories of PDOC patients, since the reduced or absent responses may be reflecting a motor damage instead.
